# ATP Induces Disruption of Tight Junction Proteins via IL-1 Beta-Dependent MMP-9 Activation of Human Blood-Brain Barrier* In Vitro*

**DOI:** 10.1155/2016/8928530

**Published:** 2016-10-04

**Authors:** Fuxing Yang, Kai Zhao, Xiufeng Zhang, Jun Zhang, Bainan Xu

**Affiliations:** ^1^Department of Neurosurgery, Chinese PLA General Hospital, 28 Fuxing Road, Haidian District, Beijing 100853, China; ^2^Department of Neurosurgery, 2nd Affiliated Hospital of Fujian Medical University, 34 Zhongshan Northern Road, Quanzhou 362000, China; ^3^Medical College, Nankai University, 94 Weijin Road, Tianjin 300071, China

## Abstract

Disruption of blood-brain barrier (BBB) follows brain trauma or central nervous system (CNS) stress. However, the mechanisms leading to this process or the underlying neural plasticity are not clearly known. We hypothesized that ATP/P2X7R signaling regulates the integrity of BBB. Activation of P2X7 receptor (P2X7R) by ATP induces the release of interleukin-1*β* (IL-1*β*), which in turn enhances the activity of matrix metalloproteinase-9 (MMP-9). Degradation of tight junction proteins (TJPs) such as ZO-1 and occludin occurs, which finally contributes to disruption of BBB. A contact coculture system using human astrocytes and hCMEC/D3, an immortalized human brain endothelial cell line, was used to mimic BBB* in vitro*. Permeability was used to evaluate changes in the integrity of TJPs. ELISA, Western blot, and immunofluorescent staining procedures were used. Our data demonstrated that exposure to the photoreactive ATP analog, 3′-O-(4-benzoyl)benzoyl adenosine 5′-triphosphate (BzATP), induced a significant decrease in ZO-1 and occludin expression. Meanwhile, the decrease of ZO-1 and occludin was significantly attenuated by P2X7R inhibitors, as well as IL-1R and MMP antagonists. Further, the induction of IL-1*β* and MMP-9 was closely linked to ATP/P2X7R-associated BBB leakage. In conclusion, our study explored the mechanism of ATP/P2X7R signaling in the disruption of BBB following brain trauma/stress injury, especially focusing on the relationship with IL-1*β* and MMP-9.

## 1. Introduction

Traumatic brain injury (TBI) often coexists with acute stress disorder (ASD) or post-traumatic stress disorder (PTSD) [1]. TBI is one of the major health problems that account for high mortality worldwide. According to the World Health Organization, TBI is expected to become the third leading cause of death and disability worldwide by 2020 [2]. TBI/PTSD or TBI/ASD are public health concerns warranting advanced investigation into the underlying mechanisms for appropriate therapeutic intervention [3]. Disruption of blood-brain barrier (BBB) is a common result following brain trauma/stress injury, and little is known about the changes in neural plasticity following brain injury and subsequent cellular responses. Currently, there is no effective neuroprotective agent that is available clinically, to prevent the damage to BBB following CNS trauma/stress injury [4, 5].

Extracellular ATP, which is dramatically increased after acute injury or stress to the central nervous system, is considered an initiator of secondary brain injury by activating a wide variety of purinergic receptors [6]. P2X7 is a unique purinergic receptor. In addition to the rapid opening of the cation channels, with prolonged exposure to high concentrations of ATP, it triggers a membrane pore facilitating the passage of large molecules, which suggests that P2X7 receptors may play a crucial role in the pathophysiology of brain trauma [7, 8]. It is reported that activation of P2X7 receptor resulted in the release of IL-1*β* [9, 10], which is an important proinflammatory cytokine. Furthermore, activation of IL-1*β* leads to the production of MMP-9 [11], which affects the tight junction proteins including ZO-1, and in turn, leads to disruption of blood-brain barrier [12, 13]. Overall, the ATP/P2X7R signaling pathway may be implicated in the process of BBB leakage following CNS trauma/stress injury. However, the underlying physiologic mechanisms following ATP-associated BBB damage and their relationship with IL-1*β* and MMP-9 are still unknown.

In this study, we used a coculture model* in vitro* comprising an immortal human cell line hCMEC/D3 expressing endothelial and BBB markers and human astrocytes to investigate the role of ATP/P2X7R signaling in the disruption of tight junction proteins [14]. We demonstrated that ATP/P2X7R signaling regulates the integrity of BBB. Activation of P2X7 receptor by ATP induces the release of IL-1*β*, which in turn enhances the production of MMP-9, leading to degradation of tight junction proteins, such as ZO-1 and occludin, resulting in the disruption of BBB.

## 2. Materials and Methods

### 2.1. Materials

Human astrocytes (HAs) were obtained from Jiamay Biolab (Beijing, China); hCMEC/D3 cell lines were purchased from Jiangyin Yuxi Biotechnology (Jiangsu, China); polyclonal anti-ZO-1 and anti-occludin antibodies were acquired from Biorbyt (Cambridge, UK, cat: orb11587, orb11181); Alexa Fluor 594-conjugated AffiniPure donkey anti-rabbit IgG was obtained from Jackson ImmunoResearch Inc. (West Grove, PA, USA, cat: 711-585-152); human interleukin-1*β* ELISA kit and human MMP-9 ELISA kit were purchased from Jiamay (Beijing, China, cat: FHK0016, FHK0144); high glucose Dulbecco's Modified Eagle's Medium (DMEM) basal medium was obtained from Hyclone (Logan, UT, United States); and EBM-2 basal medium was purchased from Lonza (Walkersville, MD, USA). Fetal bovine serum (FBS) was obtained from GIBCO (Rockville, MD, USA); phenylmethanesulfonyl fluoride (PMSF) was acquired from Amresco (Solon, OH, USA); the different cocktails were purchased from Yuanye Biotech (Shanghai, China); skimmed milk was obtained from Yili Industrial Group Co. Ltd. (Beijing, China); and anti-*β*-actin and other materials were purchased from Jiamay Biolab (Beijing, China).

### 2.2. *In Vitro* Model of Human BBB

The hCMEC/D3 cells and HAs were cultured as described in a previous study [15]. Briefly, hCMEC/D3 cells were cultured in EBM-2 basal medium supplemented with 5% FBS, growth factors, 100 U/mL of penicillin, and 100 *μ*g/mL of streptomycin. HAs were cultured in high glucose DMEM supplemented with 10% FBS, 100 U/mL of penicillin, and 100 *μ*g/mL streptomycin. Cells were maintained at 37°C in the presence of 5% CO_2_.

The contact coculture model of hCMEC/D3 and astrocytes was used as an* in vitro* model of human BBB [16]. Briefly, HAs (5 × 10^3^ cells/cm^2^) were seeded externally on Transwell inserts (polyester membranes, 6.5 mm diameter, 0.4 *μ*m pore size, Corning Costar) in an inverted position and allowed to adhere to the membrane overnight. After incubation, the hCMEC/D3 cells (2 × 10^5^ cells/cm^2^) were added to the interior of the inserts and both the cell layers were grown to confluence.

### 2.3. ELISA for IL-1*β* and MMP-9

Enzyme-linked immunosorbent assay (ELISA) was used to determine the levels of IL-1*β* and MMP-9. The hCMEC/D3 cells and HAs grown on Transwell insert were treated with A438079 (a selective P2X7 receptor antagonist, 10 *μ*M), or IL-1RA (an IL-1R antagonist, which competitively blocked the binding to IL-1 receptors, 100 *μ*g/mL), or batimastat (a MMP antagonist, 5 *μ*M) 30 min prior to the addition of BzATP (100 *μ*g/mL). The cells were exposed to photoreactive ATP analog, 3′-O-(4-benzoyl)benzoyl adenosine 5′-triphosphate (BzATP), for 2 h and their specific vehicles as control. After brief centrifugation, 100 *μ*L of cell culture supernatant samples was analyzed using human IL-1*β* ELISA kit/human MMP-9 ELISA kit according to the manufacturer's instructions. Quantitative analysis was performed on a microplate reader (Thermo fisher, Multiskan MK3).

### 2.4. Permeability of the Coculture System

The hCMEC/D3 cells and HAs were cocultured on Transwell insert. Cells were pretreated with inhibitors (A438079 10 *μ*M, or IL-1RA 100 *μ*g/mL, or batimastat 5 *μ*M) for 30 min followed by BzATP (100 *μ*g/mL) for 2 h. To characterize the changes in structural integrity of BBB models* in vitro*, we measured the permeability of the coculture system to FITC-dextran, as reported in a previous study [17]. Fluorescein isothiocyanate-labeled dextran (FITC-dextran, 10 KDa, 10 *μ*M) was added to the apical compartment. After 30 min, 100 *μ*L of the medium was removed from the basal compartment. The fluorescence of the collected samples was measured at 485 nm/520 nm using a Fluorescence Microplate Reader (Thermo Fisher, Fluoroskan Ascent FL).

### 2.5. Immunofluorescence Staining of Tight Junction Proteins

After treatment with inhibitors (A438079 10 *μ*M, or IL-1RA 100 *μ*g/mL, or batimastat 5 *μ*M) for 30 min prior to exposure to BzATP (100 *μ*g/mL) for 2 h, cells were washed twice with PBS and fixed using 4% PFA at room temperature for 30 min. Excess PFA was removed with PBS three times. The cells were permeabilized with 0.1% Triton X-100 in PBS for 10 min. After washing three times with PBS, the cells were incubated with 3% H_2_O_2_ for 10 min, followed by PBS three times. After blocking for 30 min with 10% FBS and three times with PBS, cells were incubated with polyclonal anti-occludin antibody (1 : 200) or polyclonal anti-ZO-1 antibody (1 : 200) at 4°C overnight. Unbound antibodies were removed using PBS. Alexa Fluor 594-conjugated AffiniPure donkey anti-rabbit IgG (1 : 500) was used as a secondary antibody and incubated with cells for 2 h at room temperature. Similarly, unbound antibodies were removed with PBS. Analysis and imaging were performed using a confocal laser-scanning microscope (TSC-SP2, Leica, Germany).

### 2.6. Western Blot of Tight Junction Proteins

Western blot was used to detect the expressions of occludin and ZO-1. The hCMEC/D3 cells were washed with PBS and harvested with 0.25% trypsin. The cells were suspended in 150 *μ*L ice-cold RIPA lysis buffer, sonicated 10 times for 5 s with 10 s pauses in an ice-water bath, and centrifuged at 12,000 rpm for 5 min at 4°C. Protein quantity was detected using a BCA assay kit, and equal amounts of protein were used for Western blot analysis. Next, the protein was separated by SDS-PAGE and transferred to PVDF membranes using semidry methods. Membranes were blocked in TBST + 5% skimmed milk for 2 h at room temperature. Membranes were incubated overnight at 4°C with the primary antibodies: rabbit polyclonal anti-ZO-1 antibody (1 : 100) or polyclonal anti-occludin antibody (1 : 100). The membranes were washed for 10 min with TBST buffer three times. Immunoblots were processed with secondary antibodies (1 : 2000~1 : 5000) for 1 h at room temperature. *β*-Actin was blotted on the same membrane as the internal control. Blots were quantified using Image J software (NIH).

### 2.7. Statistical Analysis

All the experiments were repeated three times and similar results were obtained. Statistical analysis was performed by one-way ANOVA (multiple comparisons) using the SPSS 16.0 statistics software (SPSS, Chicago, IL). *P* value less than 0.05 was considered statistically significant.

## 3. Results

### 3.1. A438079 Attenuated BzATP-Induced Disruption of BBB* In Vitro*

#### 3.1.1. Effect of BzATP/BzATP + A438079 on IL-1*β* and MMP-9 Induction

**Figure 1:**
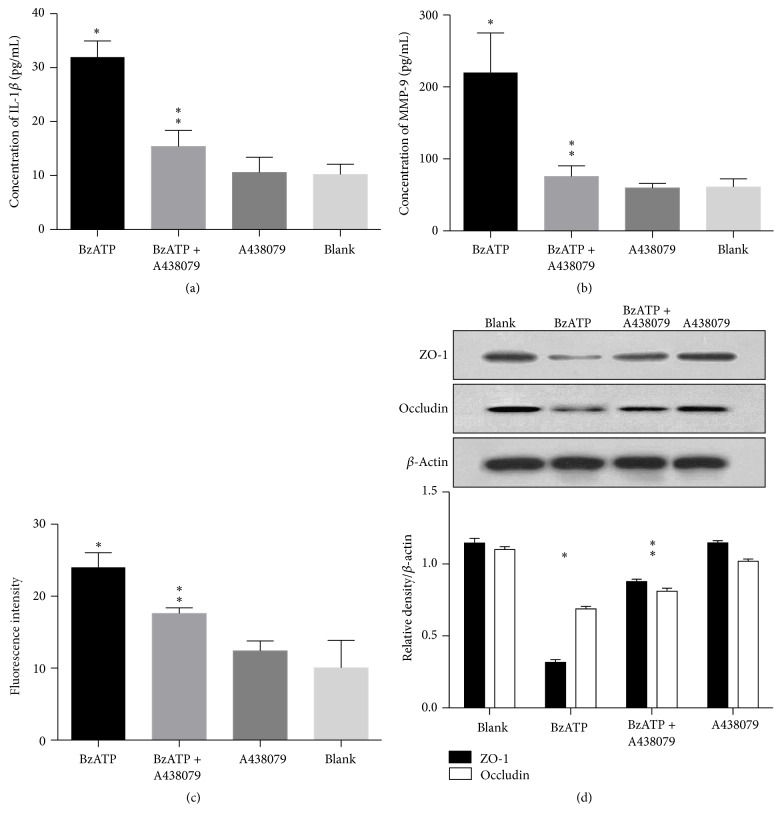
A438079 attenuates BzATP-induced disruption of blood-brain barrier* in vitro.* (a) The IL-1*β* level was significantly higher in BzATP groups than in control groups (^*∗*^ *P* < 0.05). A438079 treatment significantly reduced the levels of IL-1*β* compared with the vehicle group (^*∗∗*^ *P* < 0.05). (b) The level of MMP-9 was significantly higher in BzATP groups than in control groups (^*∗*^ *P* < 0.05). A438079 treatment significantly reduced the level of MMP-9 compared with the vehicle group (^*∗∗*^ *P* < 0.05). (c) BzATP induced hyperpermeability compared with the control groups (^*∗*^ *P* < 0.05), which was decreased by the P2X7 inhibitor A438079 in the coculture system (^*∗∗*^ *P* < 0.05). (d) Western blot analysis showed that the expression of ZO-1 and occludin in the BzATP groups was decreased compared with the controls (^*∗*^ *P* < 0.05), while A438079 treatment significantly attenuated the disruptions of ZO-1 and occludin (^*∗∗*^ *P* < 0.05).

In this study, we investigated the effects of BzATP on the TJPs of blood-brain barrier and correlated the expression of IL-1*β* and MMP-9. First, we evaluated the role of BzATP in the induction of IL-1*β* and MMP-9. ELISA results showed that the levels of IL-1*β* and MMP-9 were significantly higher in the BzATP group than in the control group without BzATP (*P* < 0.05; Figures 1(a) and 1(b)). Second, A438079 treatment significantly attenuated the increase in IL-1*β* and MMP-9, compared with the vehicle group (*P* < 0.05, Figures 1(a) and 1(b)). Furthermore, administration of A438079 alone did not significantly affect the levels of IL-1*β* and MMP-9, compared with the blank control group (*P* < 0.05, Figures 1(a) and 1(b)).

#### 3.1.2. Effect of A438079 on Permeability of Endothelial Coculture System

To determine the altered permeability of BBB model, we measured the fluorescence of FITC-dextran diffused through the coculture system. FITC-dextran (10 KDa, 10 *μ*M) diffused across BBB from the apical to the basal compartment. The fluorescence intensity of samples collected basally was measured. As shown in Figure 1(c), exposure to BzATP significantly increased permeability to FITC-dextran compared with the control group (*P* < 0.05, Figure 1(c)). Inhibition of P2X7R abolished BzATP-induced paracellular permeability, while A438079 alone did not affect basal permeability levels (*P* < 0.05, Figure 1(c)).

#### 3.1.3. Effect of A438079 on TJPs Degradation

**Figure 2:**
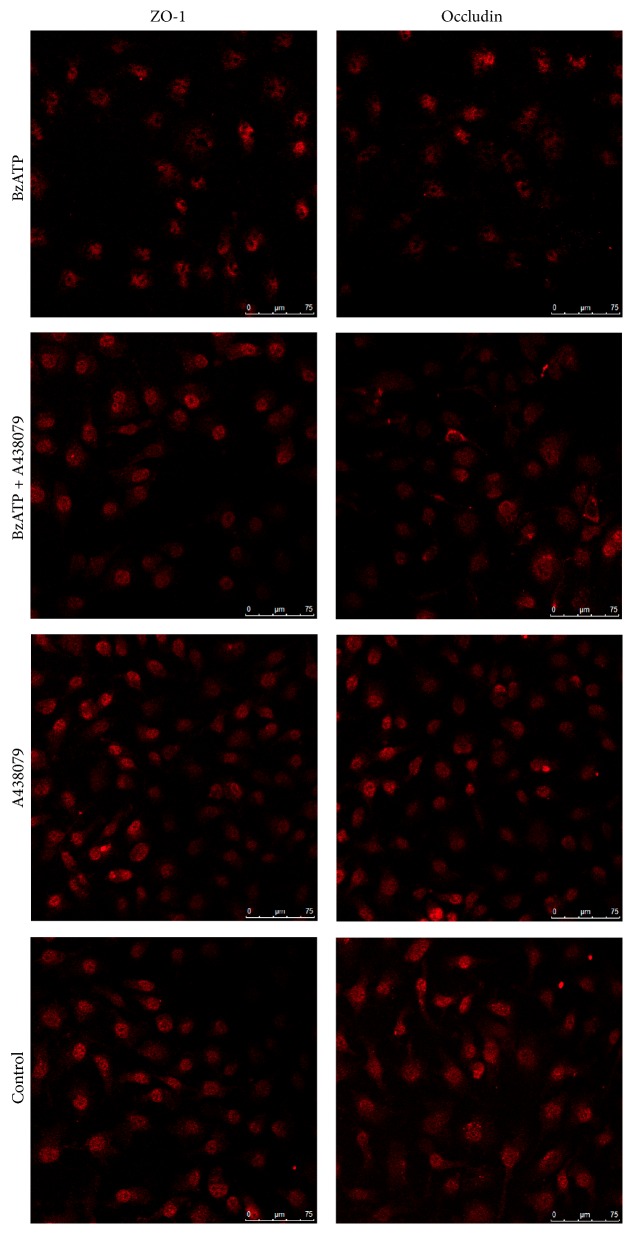
Confocal microscopy images of immunofluorescence staining of ZO-1 and occludin in hCMEC/D3 cells demonstrating the protective effect of A438079 against BzATP-induced tight junction disruption.

In an attempt to determine whether ATP-induced increase in BBB permeability was due to the altered levels of TJPs, the protein expression of occludin and ZO-1 was evaluated via immunofluorescence staining and Western blot. As shown in Figure 2, BzATP-treated cells showed significantly decreased staining of ZO-1 and occludin, which was significantly attenuated by A438079. Furthermore, Western blot analysis confirmed the decrease in the expression of ZO-1 and occludin in BzATP groups compared with the controls. Treatment with A438079 enhanced the levels of ZO-1 and occludin compared with BzATP alone (*P* < 0.05, Figure 1(d)). The results of Western blot were similar to immunofluorescence staining of occludin and ZO-1 in the following experiments.

### 3.2. IL-1*β* Inhibition Attenuated BzATP-Induced Tight Junction Disruption and Endothelial Coculture Hyperpermeability

#### 3.2.1. BzATP-Induced MMP-9 Induction Was Attenuated by IL-1RA

**Figure 3:**
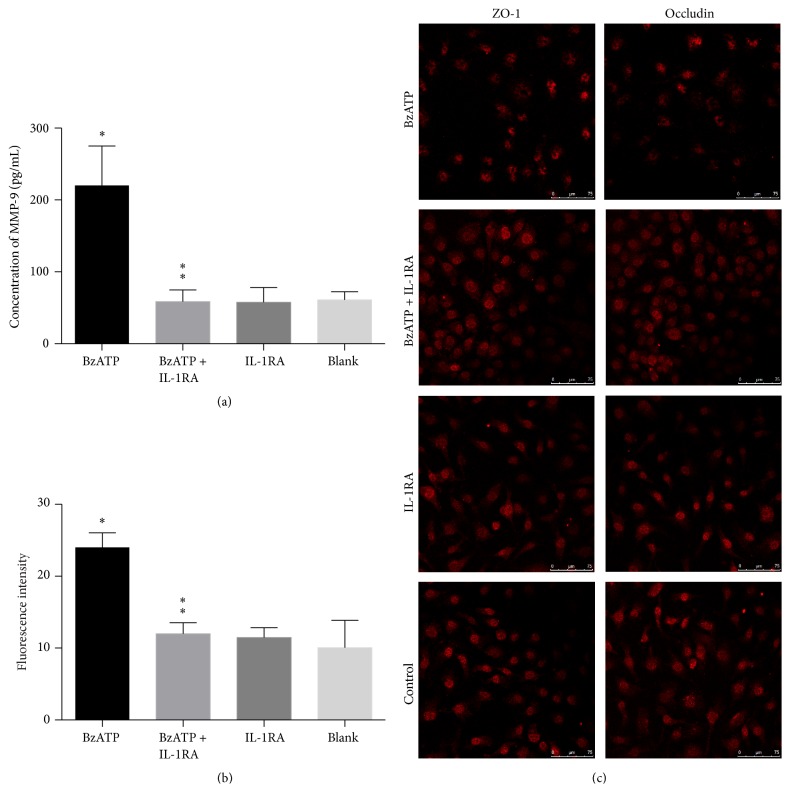
BzATP-induced tight junction disruption and hyperpermeability of endothelial coculture system were attenuated by IL-1*β* inhibition. (a) The levels of MMP-9 were significantly higher in the BzATP groups than in the control groups (^*∗*^ *P* < 0.05). IL-1RA treatment significantly reduced the level of MMP-9 versus vehicle group (^*∗∗*^ *P* < 0.05). (b) BzATP induces hyperpermeability versus control groups (^*∗*^ *P* < 0.05), which was decreased by IL-1RA in the coculture system (^*∗∗*^ *P* < 0.05). (c) Confocal microscopy images of immunofluorescence staining of ZO-1 and occludin demonstrating the protective effect of IL-1RA against BzATP-induced tight junction disruption.

As discussed in Section 3.1.1, BzATP induced the release of IL-1*β* and MMP-9 in the BBB model. In order to correlate IL-1*β* and MMP-9, we designed two experiments (Sections 3.2 and 3.3), using their inhibitors, respectively. First, we investigated whether IL-1RA inhibited the BzATP-induced increase in MMP-9. As shown in Figure 3, IL-1RA treatment significantly attenuated the increase in MMP-9 levels triggered by BzATP compared with the vehicle group (*P* < 0.05, Figure 3(a)).

#### 3.2.2. IL-1RA Attenuated Hyperpermeability of BzATP-Induced Endothelial Coculture System

Exposure to BzATP significantly increased the permeability to FITC-dextran compared with control group (*P* < 0.05, Figure 3(b)). IL-1RA attenuated BzATP-induced paracellular permeability. However, IL-1RA alone did not affect basal permeability levels (*P* < 0.05, Figure 3(b)).

#### 3.2.3. BzATP-Induced TJP Disruption Was Attenuated by IL-1RA

Immunofluorescence staining showed that the expression of ZO-1 and occludin in BzATP groups was significantly decreased compared with controls, while treatment with IL-1RA and BzATP significantly increased the levels of the two tight junction proteins, indicating that the treatment reversed the degradation of TJPs (Figure 3(c)).

### 3.3. BzATP-Induced Tight Junction Disruption and Hyperpermeability of the Endothelial Coculture System Were Attenuated by MMP

#### 3.3.1. BzATP-Induced IL-1*β* Induction Was Not Attenuated by MMP-9 Inhibition

**Figure 4:**
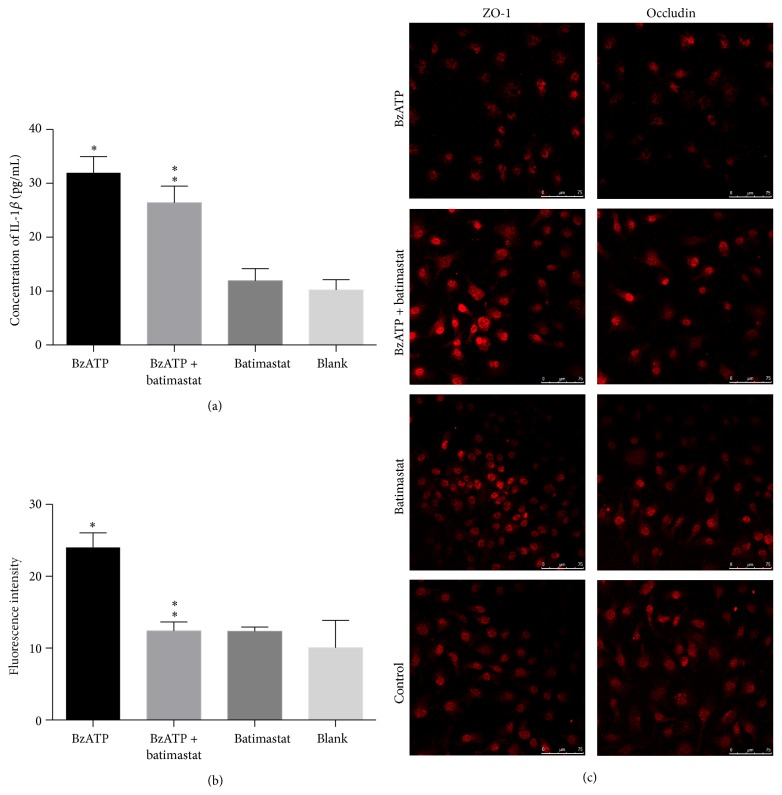
MMP-9-induced tight junction disruption and endothelial coculture system hyperpermeability were not attenuated by IL-1*β* inhibition. (a) The levels of IL-1*β* were significantly higher in BzATP groups than in control groups (^*∗*^ *P* < 0.05). However, batimastat + BzATP treatment did not alter the level of IL-1*β* versus BzATP group (^*∗∗*^ *P* > 0.05). (b) Exposure to BzATP significantly increased the permeability to FITC-dextran compared with the control groups (^*∗*^ *P* < 0.05). Batimastat attenuated BzATP-induced paracellular permeability versus BzATP group (^*∗∗*^ *P* < 0.05). (c) Confocal microscopy images of immunofluorescence staining of ZO-1 and occludin demonstrating the protective effect of batimastat against BzATP-induced tight junction disruption.

ELISA showed that the levels of IL-1*β* were significantly higher in the BzATP-treated groups than in the control group without BzATP. However, batimastat treatment did not alter the level of IL-1*β* (*P* > 0.05, Figure 4(a)).

#### 3.3.2. MMP Inhibition Attenuated Hyperpermeability of BzATP-Induced Endothelial Coculture System

Exposure to BzATP significantly increased the permeability to FITC-dextran compared with the control group (*P* < 0.05, Figure 4(b)). Batimastat attenuated BzATP-induced paracellular permeability, while batimastat alone did not affect the basal permeability levels (*P* < 0.05, Figure 4(b)).

#### 3.3.3. BzATP-Induced TJP Disruption Was Attenuated by MMP Inhibition

Immunofluorescence staining showed that the expression of ZO-1 and occludin in the BzATP group was significantly decreased compared with that of the controls. In contrast, treatment with batimastat combined with BzATP significantly increased the levels of the two tight junction proteins, indicating that batimastat treatment reversed the degradation of TJPs (Figure 4(c)).

### 3.4. IL-1*β*-Induced Tight Junction Disruption and Hyperpermeability of Endothelial Coculture System Were Attenuated by MMP-9 Inhibition

#### 3.4.1. IL-1*β*-Induced MMP-9 Induction Was Attenuated by MMP-9 Inhibition

**Figure 5:**
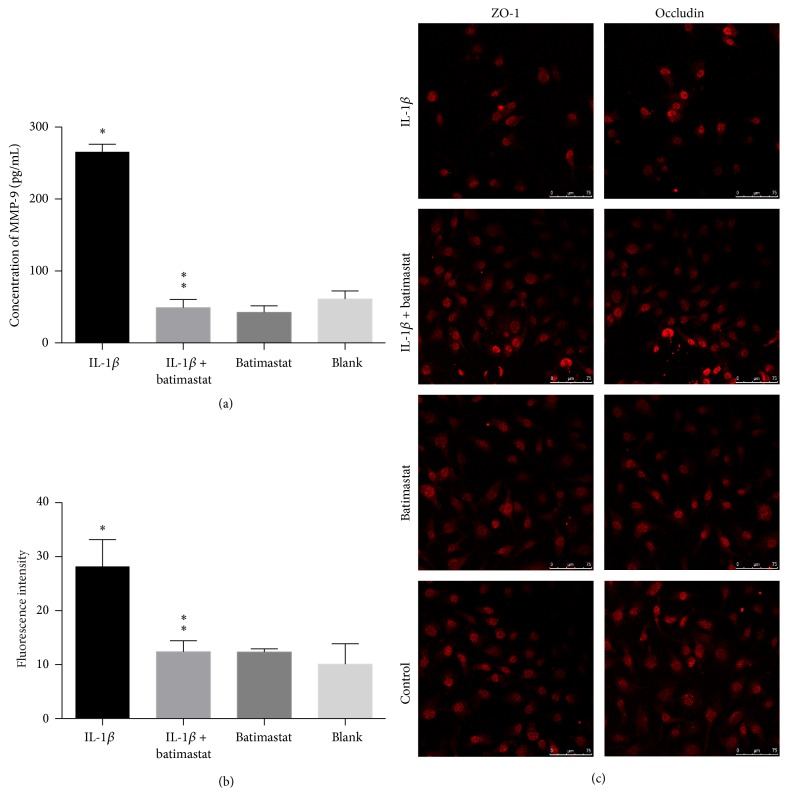
IL-1*β*-induced tight junction disruption and endothelial coculture system hyperpermeability were attenuated by MMP-9 inhibition. (a) The levels of MMP-9 were significantly higher in IL-1*β* groups than in control groups (^*∗*^ *P* < 0.05). Batimastat treatment significantly reduced the level of MMP-9 versus vehicle group (^*∗∗*^ *P* < 0.05). (b) Exposure to IL-1*β* significantly increased the permeability to FITC-dextran compared with the control groups (^*∗*^ *P* < 0.05), which was decreased by batimastat in coculture system (^*∗∗*^ *P* < 0.05). (c) Confocal microscopy images of immunofluorescence staining of ZO-1 and occludin demonstrating the protective effect of batimastat against IL-1*β*-induced tight junction disruption.

We investigated the association between IL-1*β* and MMP-9 in the absence of BzATP. Our study showed that IL-1*β* treatment significantly increased the levels of MMP-9, compared with the vehicle group (*P* < 0.05, Figure 5(a)). Apparently, administration of batimastat significantly attenuated the levels of MMP-9 (*P* < 0.05, Figure 5(a)). However, MMP-9 did not significantly affect the level of IL-1*β*, as discussed in Section 3.5.1.

#### 3.4.2. MMP-9 Inhibition Attenuated Hyperpermeability of IL-1*β*-Induced Endothelial Coculture System

Exposure to IL-1*β* significantly increased the permeability to FITC-dextran compared with control group (*P* < 0.05, Figure 5(b)). Batimastat attenuated IL-1*β*-induced paracellular permeability, while batimastat alone did not affect the basal permeability levels (*P* < 0.05, Figure 5(b)).

#### 3.4.3. IL-1*β*-Induced TJP Disruption Was Attenuated by MMP-9 Inhibition

Immunofluorescence staining showed that the expression of ZO-1 and occludin in the IL-1*β* groups was significantly decreased compared with the controls, while batimastat + IL-1*β* groups showed a significant increase in the levels of the two tight junction proteins, indicating that batimastat treatment reversed the degradation of TJPs (Figure 5(c)).

### 3.5. MMP-9-Induced Tight Junction Disruption and Hyperpermeability of Endothelial Coculture System Were Not Attenuated by IL-1*β* Inhibition

#### 3.5.1. MMP-9 Has No Effect on IL-1*β* Induction

**Figure 6:**
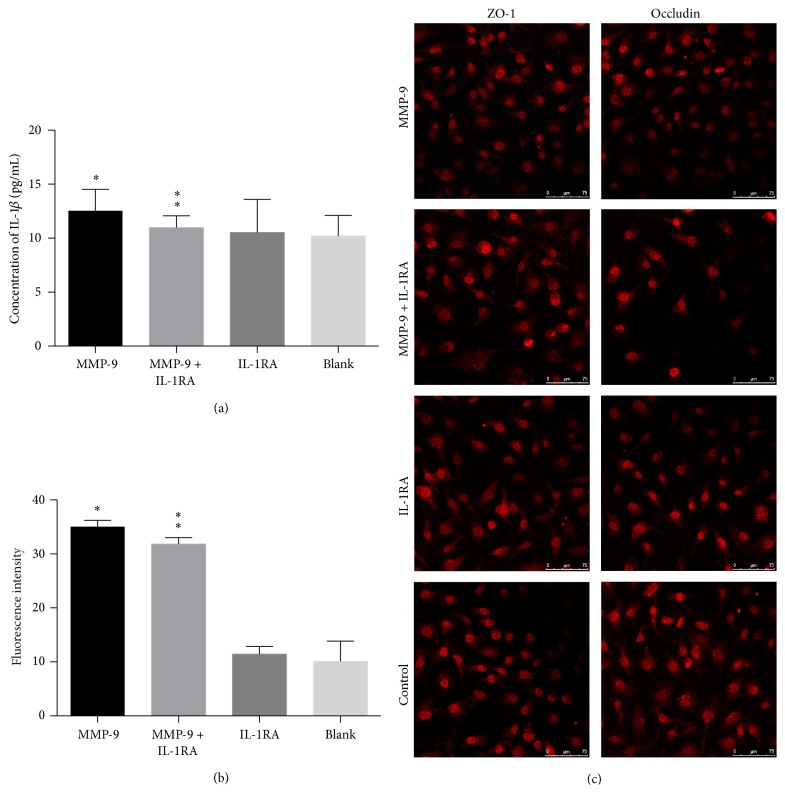
BzATP-induced tight junction disruption and endothelial coculture system hyperpermeability were attenuated by MMP inhibition. (a) The levels of IL-1*β* were not significantly altered in MMP-9-treated groups versus control groups (^*∗*^ *P* > 0.05). No statistically significant differences were found between IL-1RA + MMP-9 groups and MMP-9 groups in the levels of IL-1*β* (^*∗∗*^ *P* > 0.05). (b) Exposure to MMP-9 significantly increased the permeability to FITC-dextran compared with the control group (^*∗*^ *P* < 0.05). However, IL-1RA failed to attenuate MMP-9-induced paracellular hyperpermeability (^*∗∗*^ *P* > 0.05). (c) Immunofluorescence staining showed that the expression of ZO-1 and occludin in MMP-9 groups was significantly decreased compared with controls, while IL-1RA + MMP-9 groups showed no improvement in the levels of the two tight junction proteins.

To further confirm the relationship between IL-1*β* and MMP-9, we investigated whether MMP-9 elevated the levels of IL-1*β*. ELISA analysis showed that the levels of IL-1*β* were not altered in MMP-9-treated groups compared with controls. In addition, there were no significant differences in the levels of IL-1*β* between IL-1RA + MMP-9 and MMP-9 groups (*P* > 0.05, Figure 6(a)).

#### 3.5.2. Hyperpermeability of MMP-9-Induced Endothelial Coculture System Was Not Attenuated by IL-1*β* Inhibition

Exposure to MMP-9 significantly increased the permeability to FITC-dextran compared with the control group (*P* < 0.05, Figure 6(b)). However, IL-1RA failed to attenuate MMP-9-induced paracellular hyperpermeability (*P* < 0.05, Figure 6(b)).

#### 3.5.3. MMP-9-Induced TJP Disruption Was Not Attenuated by IL-1*β* Inhibition

Immunofluorescence staining showed that the expression of ZO-1 and occludin in the MMP-9 groups was significantly decreased compared with that of the controls. However, the IL-1RA + MMP-9 groups showed no improvement in the levels of the two tight junction proteins, indicating that IL-1RA treatment failed to reverse the degradation of TJPs (Figure 6(c)).

## 4. Discussion

Disruption of BBB is a hallmark of brain trauma/stress injury. It is associated with an increase in the permeability of damaged endothelium leading to cerebral edema [4]. The structural integrity of BBB is determined by tight junction proteins (TJPs). In other words, the decreased expression of TJPs in the brain microvessels is strongly correlated with disruption of the BBB [18, 19]. In addition, evidence strongly suggests that P2X7 receptors are activated by extracellular ATP following CNS injury/stress, triggering secondary brain damage [6], and deficits in the structural integrity of the tight junction proteins, leading to BBB dysfunction and brain edema [4, 20]. Therefore, elucidation of the cellular mechanisms of ATP-associated BBB damage may facilitate the development of effective therapeutics to improve patient outcomes after brain injury.

The increasingly important role of P2X7R in the pathophysiology of CNS trauma/stress suggests that targeted therapies hold the key to the treatment of cerebral edema following CNS stress/trauma. Previous reports showed that administration of BBG, an antagonist of P2X7 receptor, improved neurological function and exerted neuroprotective effect in spinal cord and traumatic brain injuries in murine models [21–23]. However, activation of P2X7R-induced disruption of tight junction proteins in human BBB models has not been reported. Therefore, we utilized an* in vitro* coculture BBB model consisting of an immortal human cell line hCMEC/D3 with human astrocytes to investigate the role of ATP/P2X7R signaling in the disruption of tight junction proteins. Our model extends previous work in determining the translational potential of human cells. In the present study, we demonstrated that A438079, a more selective antagonist of P2X7 receptors, attenuated hyperpermeability in a human BBB model as well as disruption of ZO-1 and occludin, which is closely associated with IL-1*β* and MMP-9 expression.

P2X7R is the receptor mediating ATP-dependent maturation and release of IL-1*β*. Activation of caspase-1 plays a key role in P2X7R-dependent IL-1*β* release. ATP/P2X7R signaling leads to the opening of large membrane pores, triggering changes in the intracellular ionic homeostasis to activate the caspase-1 cascade resulting in the transformation of pro-IL-1*β* to IL-1*β* [9]. Exposure to Ac-YVAD-CMK, a caspase-1 antagonist, attenuated the maturation of IL-1*β* induction and inhibited MMP-9 activation and conservation of ZO-1 in mice [24]. Using a human BBB model* in vitro*, we confirmed that the extracellular ATP-induced activation of P2X7R led to the release of IL-1*β*, which was inhibited by the administration of A438079. Consistent with pharmacological inhibition of P2X7R, a significant reduction in IL-1*β* expression following experimental brain trauma/stress was observed in the cortex of P2X7 knockout mice, when compared with wild-type mice [22]. In addition, treatment with an IL-1 receptor antagonist followed by BzATP significantly attenuated the disruption of ZO-1 and occludin, indicating that ATP/P2X7R signal-induced degradation of tight junction proteins was strongly associated with IL-1 receptors. Overall, our study suggested that secretion of IL-1*β* induced by ATP-stimulated P2X7R activation played a crucial role in triggering endothelial hyperpermeability and dysfunction of BBB.

In the present study, we investigated the relationship between IL-1*β* and MMP-9. IL-1*β*, one of the proinflammatory cytokines, is an important mediator of brain injuries/stress, and its role in the induction of MMP-9 has been well documented [24–26]. Ranaivo et al. reported that IL-1*β* enhanced the release of MMP-9 via activation of the ERK pathway [27]. However, Wu and his colleagues argued that IL-1*β*-induced MMP-9 expression depends on JNK1/2 signaling in astrocytes, involving CaMKII and phosphorylation of c-Jun, which are the upstream and downstream regulators of JNK, respectively [28, 29]. In our study, we found that both IL-1*β* and MMP-9 were induced after ATP/P2X7R activation. Induction of IL-1*β* increased the level of MMP-9. However, the levels of IL-1*β* were not altered in MMP-9-treated groups. Therefore, no obvious interaction exists between MMP-9 and IL-1*β* following disruption of BBB secondary to brain trauma/stress.

MMPs comprise a number of zinc-dependent endopeptidases that mediate the degradation of extracellular matrix. MMP-9 has a significant role in CNS injury/stress following activation by IL-1*β* and close association with tight junction protein degradation [30]. ATP-treated glial cultures derived from neonatal C57BL/6 mice showed an increase in MMP-9 activity via activation of the P2X7 receptor [31]. In addition, increased MMP-9 activity promoted the disruption of tight junctions in cerebral endothelial cells, such as occludin and ZO-1, which in turn disrupt the structural integrity and hyperpermeability of BBB [12, 32, 33]. Pharmacological inhibitors of MMP-9 significantly attenuated the disruption of BBB in rats [34]. Similarly, MMP-9 knockout abrogated the increased vascular permeability and restored neurological function [35, 36]. Our study showed that inhibitors of P2X7R and IL-1*β* prevented the increase in MMP-9 expression and consequently suppressed the degradation of ZO-1 and occludin in human BBB model* in vitro*. Furthermore, inhibition by batimastat decreased the disruption of tight junction proteins, indicating that MMP-9 was essential for ATP-induced hyperpermeability of BBB. These findings strongly supported the hypothesis that MMP-9 was regulated upstream by IL-1*β* and modulated BBB integrity downstream following CNS trauma/stress, suggesting that MMP-9 may also represent a therapeutic target as well as IL-1*β* inhibition in the ATP-induced BBB leakage.

Our study has several limitations. First, BBB model* in vitro* does not simulate the* in vivo* conditions, totally. For example, shear stress played a role in BBB physiology* in vivo* [37]. Second, studies suggest that microglia are the main cell type in the brain, which is responsible for IL-1*β* and IL-18 secretion [38]. Therefore, analysis of the relationship between microglia and other cell types such as astrocytes is critical to fully understand the mechanism of ATP/P2X7R signaling. Third, BBB disruption is a complex and multistep process involving several interrelated factors. As a result, agents such as P2X7R antagonists that selectively target specific steps in the process are likely to be more effective when deployed in combination with agents that target other processes [39].

## 5. Conclusions

Our study demonstrated the potential mechanism of ATP/P2X7R signaling that may be targeted to attenuate BBB disruption and brain edema following brain trauma/stress. Concurrently, we tested P2X7R antagonist in a human BBB model, indicating its translational potential. However, a more detailed understanding of ATP/P2X7R pathway is needed. Future studies will focus on elucidating the mechanisms of cytokine-mediated neural plasticity and their implications for the pathophysiology and treatment of brain trauma/stress.

## Abbreviations

BzATP: 3′-O-(4-Benzoyl)benzoyl adenosine 5′-triphosphate
BBB: Blood-brain barrier
CNS: Central nervous system
P2X7R: P2X7 receptor
IL-1*β*: Interleukin-1*β*
MMP-9: Matrix metalloproteinase-9
TJP: Tight junction protein
ELISA: Enzyme-linked immunosorbent assay
IL-1R: Interleukin-1 receptor
IL-1RA: Interleukin-1 receptor antagonist
TBI: Traumatic brain injury
ASD: Acute stress disorder
PTSD: Post-traumatic stress disorder
HA: Human astrocyte
DMEM: High glucose Dulbecco's Modified Eagle's Medium
FBS: Fetal bovine serum
PMSF: Phenylmethanesulfonyl fluoride
FITC: Fluorescein isothiocyanate.
